# Comparison of Abdominal Muscle Thickness between the Abdominal Draw-in Maneuver and Maximum Abdominal Contraction Maneuver

**DOI:** 10.3390/healthcare10020251

**Published:** 2022-01-28

**Authors:** Seo-Yoon Park, Seunghue Oh, Ki-Hyun Baek, Sung-Soo Bae, Jung-Won Kwon

**Affiliations:** 1 Department of Physical Therapy, College of Health Sciences, Dankook University, Cheonan 31116, Korea; pgy0614@hanmail.net; 2 Department of Physical Therapy, Youngnam University College, Daegu 42415, Korea; rock3224@naver.com; 3 Department of Health, Graduate School, Dankook University, Cheonan 31116, Korea; backho86@naver.com; 4 Department of Physical Therapy, College of Rehabilitation Science, Daegu University, Gyeongsan 38453, Korea; definikk@gmail.com

**Keywords:** core stability, abdominal muscle thickness, breathing exercise, maximum expiration, abdominal draw-in maneuver

## Abstract

All abdominal muscles, including the transverse abdominis (TrA), should be modulated to improve core stability. This study aimed to investigate easier and more effective core exercise methods by comparing thickness changes in the TrA, internal oblique (IO), external oblique (EO), and rectus abdominis (RA) muscles during the abdominal draw-in maneuver (ADIM) and maximum abdominal contraction maneuver (MACM). Thirty healthy subjects who participated in this study underwent ADIM and MACM three times in random order. We measured the abdominal muscle thickness during ADIM and MACM using ultrasonography and compared the changes in the thickness of TrA, IO, EO, and RA muscles using a paired *t*-test. Significant differences were observed in the thicknesses of all the abdominal muscles between the ADIM and MACM groups (*p* < 0.05). The MACM immediately increased the thickness of the TrA (*p* < 0.001, effect size (ES) = 0.931), IO (*p* = 0.001, ES = 0.761), EO (*p* = 0.008, ES = 0.415), and RA (*p* < 0.001, ES = 0.767) muscles. These results suggest that MACM is useful for immediately increasing the thickness of TrA, IO, EO, and RA muscles and may contribute to the clinical effect of simultaneous contractions on the changes in abdominal muscle thickness.

## 1. Introduction

Core stability is the cooperative contraction and coordination modulation of a group of muscles in the body, particularly the abdomen in the front, the spine muscles at the back, the diaphragm on top, and the pelvic floor muscles at the bottom [1,2]. Small and deep core muscles, as well as large and superficial core muscles, are required for optimal core function and should be contracted with appropriate tension and timing [3,4]. The transverse abdominis (TrA), internal oblique (IO), external oblique (EO), and rectus abdominis (RA) muscles play important roles in core stability during body movements and weight bearing [4,5]. However, previous studies have found that the TrA and IO muscles are not activated separately and that simultaneous contraction occurs [6,7]. Therefore, all abdominal muscles, including the TrA, should be regulated to improve core stability [8,9,10].

The abdominal draw-in maneuver (ADIM), bridging exercise, curl-up, abdominal bracing, and maximum expiration are core exercises that develop and maintain core stability [8]. Of these, the ADIM is a common exercise method that can be used to assess TrA function [8,11]. In addition, the ADIM promotes selective contraction of the transverse muscles while maintaining minimum contraction of the synergistic muscles, such as the IO, EO, RA, and erector spinae [12,13]. However, it is difficult to educate patients on how to correctly perform the ADIM; patients often have difficulty using other trunk muscles or regulating their breathing [7]. As an alternative exercise method, maximum expiration has been used to facilitate core stability, which provides a co-contraction of deep and superficial abdominal muscles by acting as active functional breathing muscles [4,14]. In addition, maximum expiration induces activation of the pelvic floor muscles, which helps stabilize the core by activating deep abdominal and paravertebral muscles and increasing abdominal pressure [15,16]. Co-contraction of these abdominal muscles due to maximum expiration has greater benefits than the ADIM in terms of improving lumbar stabilization, because all abdominal muscles contribute to core stability [17]. Previous studies have also suggested that a combination of maximum expiration and core stabilization exercises can more effectively activate all abdominal muscles [4,17,18].

Based on these findings, we designed a maximum abdominal contraction maneuver (MACM) consisting of maximum expiration and lower limb movement. The MACM focuses on maximal co-contraction of all the abdominal muscles by contracting a hip joint adductor muscle with maximum expiration in the hook-lying position. The MACM also increases abdominal muscle thickness, especially in the TrA and RA muscles, more than maximum expiration alone [19,20]. Therefore, the MACM, which combines maximum expiration and lower limb movement, can induce co-contractions of superficial and deep abdominal muscles, as well as selective muscle contraction. However, only one study has investigated changes in the thicknesses of all the abdominal muscles after the MACM compared with maximum expiration alone [19]. Several exercise methods, such as the ADIM, maximum expiration, and bridge exercise, have been studied to improve core stability, but few have studied MACM that use maximum expiration with hip adduction [4,5,8]. The abdominal muscles serve as an important component of the core and are the most powerful expiration muscles. During resting ventilation, the abdominal muscles are recruited in a different sequence: the TrA is the most active, the IO and EO are of intermediate activity, and the rectus abdominis muscle is the least active expiratory muscle [21,22]. Therefore, specific and isolated exercises for superficial and deep muscle strengthening are required. However, the effect of the MACM compared with that of the ADIM on muscle thickness has not been previously investigated.

Therefore, this study aimed to compare the thickness changes of the TrA, IO, EO, and RA muscles after the ADIM and MACM and investigated their immediate effect on core stability in healthy adults.

## 2. Methods

### 2.1. Participants

This was a cross-sectional study 30 healthy subjects (17 males and 13 females) participated in. The general characteristics of the subjects are shown in Table 1. An a priori power analysis indicated that a sample size of 30 was needed (a moderate effect size (ES) of 0.5, an α-error of 0.025, and a power of 0.95) [23]. The inclusion criteria were as follows: (1) the ability to breathe without pain and fully adduct hips, (2) understanding the exercise method and following the experimental procedures, (3) no history of spine or pelvic pain or dysfunction, (4) no history of hip or spinal injury or surgery, (5) no history of rib fracture, and (6) no respiratory disease or not taking any medications. The exclusion criteria were a history of low back pain, known neuromuscular disease, and women with a history of pregnancy. Prior to the study, all subjects were informed about the experiment and voluntarily agreed to participate. The experiment was conducted with the approval of the Institutional Review Board (IRB, 2019-11-016-002; date: 30 December 2019).

### 2.2. Materials

An 8-MHz linear transducer ultrasound instrument (SonoAce R7, Samsung Medison Co., Seoul, Korea) with proven reliability and validity in clinical and research settings was used as the measurement tool [24]. The linear transducer was positioned horizontally to the right of the subject’s body at the time of measurement. The thickness of the TrA, IO, and EO muscles was measured in a direction parallel to the muscle fibers of the transverse abdominal muscle at 25-mm anteromedial at the halfway point between the right axillary and upper midline iliac crest and the 12th rib inferior angle [25,26]. When measuring the RA muscle thickness, the linear transducer was placed 25 mm next to the navel. The thicknesses of the TrA, IO, EO, and RA muscles were measured using a caliper built into the ultrasound machine and the distance between fasciae. The muscles were measured by drawing a vertical line 10 mm above the horizontal line from the corner of the inner aponeurosis of the transverse abdominal muscles [27]. RA thickness was measured by placing the transducer 2 to 3 cm above the umbilicus and 2 to 3 cm from the midline, defined as the longest length in the vertical direction of the muscle from the center of the ultrasound image [28]. Measurements were made by an experienced physiotherapist to minimize interpreter differences. The order of measurements was randomized, and the measurements were repeated on the same day for interrater reliability.

### 2.3. Procedure

**Figure 1:**
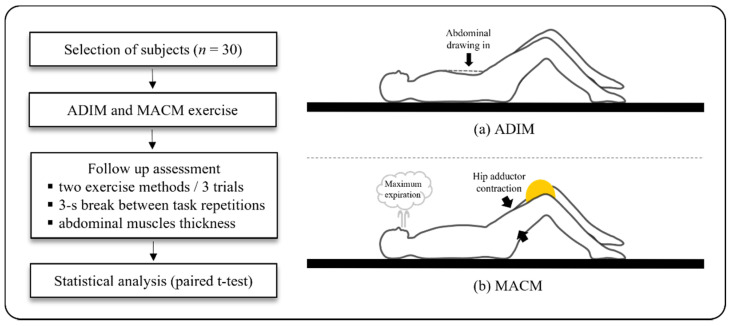
Flowchart of the experimental protocol and the two maneuvers. (**a**) ADIM: abdominal draw-in maneuver and (**b**) MACM: maximum abdominal contraction maneuver.

All subjects performed the ADIM and MACM in random order (Figure 1). Both the ADIM and MACM were performed on separate days with a 1-week interval. The subjects were trained for 15 min to learn the ADIM and MACM before the experiment [7]. Then, the subjects were asked to place their arms on both sides of the bed parallel to the trunk in the supine position. The ADIM and MACM were performed as follows: (1) For the ADIM, the subject lied in the supine position, with both arms parallel to the trunk, hip at 45° flexion, and knee joint at 90°. The subject was instructed to “exhale and pull your navel to move up and back without pelvic movement” [7,29]. (2) For the MACM, the subject lied in the supine position with both arms parallel to the trunk, hip at 45° flexion, and knee joint at 90°. Then, a 22-cm diameter Redondo ball was placed between the knees of the subject to achieve contraction of the hip adductor muscles. The subject was asked to squeeze the ball as much as possible between both knees during maximum expiration. The subject was instructed to “exhale as much as you can for about 10 s without a brace pattern from the end of maximum expiration” [20,30].

A total of 12 ultrasonographic images were captured in both the resting and active phases: three at the end of resting expiration before the ADIM, three at end of resting expiration before the MACM, three at end of active expiration during the ADIM, and three at the end of active expiration during the MACM. Images were captured three times for each abdominal muscle, namely the TrA, IO, EO, and RA muscles. Furthermore, while performing the task, the ultrasound screen was hidden, since the subjects were blinded to the procedure. To prevent muscle fatigue during measurements, a 3-s break was allowed between task repetitions (Figure 1). The average data from the three trials were used for data analysis.

### 2.4. Data Analysis

**Figure 2:**
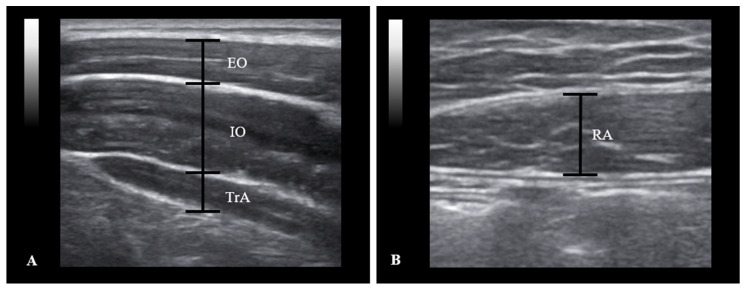
Abdominal muscle ultrasound image obtained in the right horizontal position. (**A**) TrA: transverse abdominis; IO: internal oblique; EO: external oblique; (**B**) RA: rectus abdominis.

SPSS 22.0 for Windows (IBM Inc. Chicago, IL, USA) was used for statistical analysis of the experiment. All data were presented as the mean and standard deviation. On-screen calipers were used to measure the thickness of each muscle. The EO, IO, and TrA thicknesses were measured by drawing a perpendicular line 1 cm from the edge of the aponeurosis of the abdominal muscles (Figure 2A). The RA thickness was determined as the longest length of muscle drawing a perpendicular line from the center of the ultrasound image (Figure 2B). The thickness of each abdominal muscle was measured three times, and the average value was calculated. The change in muscle thickness was calculated by subtracting the value of the resting phase from the value of the active phase. A Shapiro–Wilk test was used to determine the normal distribution for the data of each subject. A paired *t*-test was performed to compare the muscle thickness changes in the resting and active phases of the TrA, IO, EO, and RA muscles during the ADIM and MACM. Cohen’s d and 95% confidence intervals (CI) were calculated to determine the within-group ES. The ES was categorized as large (≥0.80), moderate (≥0.50), or small (≥0.20) [31]. *p*-values < 0.05 were considered statistically significant.

## 3. Results

**Figure 3:**
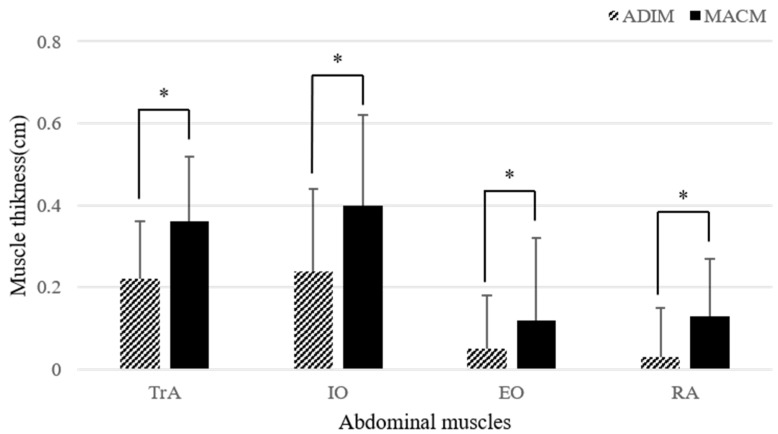
Comparison of the changes in abdominal muscle thickness. ADIM: abdominal draw-in maneuver; MACM: maximum abdominal contraction maneuver; TrA: transverse abdominis; IO: internal oblique; EO: external oblique; RA: rectus abdominis. * *p* < 0.05.

The mean age, height, weight, and body mass index of the subjects were 23.03 ± 1.73 years, 168.37 ± 7.80 cm, 65.67 ± 13.58 kg, and 23.01 ± 3.50 kg/m^2^, respectively (Table 1). The muscle thickness changes of the TrA, IO, EO, and RA muscles are shown in Table 2. The MACM significantly increased the thickness of the TrA, IO, EO, and RA muscles compared to that in the ADIM (*p* < 0.05) (Figure 3).

The ES for the muscle thickness changes of the TrA, IO, EO, and RA muscles were determined from the pooled standard deviation measures. A large effect was identified for the TrA muscle (0.931), a moderate effect for both the IO (0.761) and RA muscles (0.767), and a small effect for the EO muscle (0.415) (Table 2).

## 4. Discussion

This study was conducted to quantify the changes in the thicknesses of all the abdominal muscles between the resting and active phases during the ADIM and MACM. We found that the MACM significantly increased the thickness of the TrA, IO, EO, and RA muscles compared to that in the ADIM. These results suggest that the MACM is more favorable in increasing core stability and abdominal pressure, and its superiority is attributed to the increased thicknesses of all the abdominal muscles, probably caused by hip adduction accompanied by abdominal muscle co-contraction. Previous studies reported that core exercise combined with lower limb movement enhances abdominal muscle co-contraction [7,32,33]. In addition, the co-contraction of ipsilateral and contralateral hip joint adductors and abductors has been shown to help improve pelvic balance and trunk stability [15,34]. These results are consistent with our findings that the MACM with additional lower limb movement can induce co-contraction of all the abdominal muscles. Bridge exercises using a ball between the knee joints activate the hip adductor muscle, which causes hip adduction and internal rotation, and induce co-contraction of the trunk and pelvic floor muscles such as the TrA and RA [35]. In addition, Jang et al. reported that performing hip adduction increased the activities of the EO, IO, and RA muscles during the bridging exercise in healthy female adults [36]. They suggested that the greater muscle activity during the bridging exercise with hip adduction affects the control of the trunk muscles attached to the pelvis because the hip adductors originate proximal to the inferior aspect of the body and ischium and insert distally on the femur. Hip adductors are linked to the trunk muscles to support core stability and play an important role in the contraction of the abdominal and pelvic floor muscles [7,19]. Thus, hip adductor contraction during the MACM leads to increased abdominal muscle activity and contributes to core stability.

In this study, greater muscle thickness in all the abdominal muscles after the MACM suggests that maximum expiration may exhibit stronger co-contraction than that in the ADIM. When forced expiration or coughing occurs, the anterior and lateral abdominal muscles contract, causing pressure to move the diaphragm upward. After pressure develops, the pelvic floor muscles contract and maintain abdominal pressure [37]. Pelvic floor muscle contraction increases the abdominal pressure to provide core stability and induces the co-contraction of deep abdominal muscles during breathing [16,17]. In addition, the role of the pelvic floor muscle is essential for the synergy of the diaphragm and abdominal muscles to maintain the intra-abdominal pressure [17]. Thus, it seems that co-contraction of all the abdominal muscles by the MACM increases the activity of the pelvic floor muscles and intra-abdominal pressure and that more strenuous breathing efforts might increase the thickness of the muscles immediately after the MACM compared with that after the ADIM. For this reason, the MACM was designed with additional lower limb movement that focuses on the co-contraction of abdominal muscles.

This study confirmed that the MACM can result in further co-contraction of all the abdominal muscles compared to that in the ADIM, which suggests that it can overcome the limitations of the ADIM. It is easier to educate patients with the MACM than the ADIM, and no difficulties were encountered when substituting different trunk synergists or regulating patients’ breathing [7]. However, this study had several limitations. First, our results cannot be generalized to other populations, because all the subjects were healthy adults. Second, the evaluators who recorded the ultrasonographic images were not blinded to the study. However, the operator who analyzed the images was blinded by coding the patients prior to the image measurements. Third, it was difficult to determine the duration of the persistence of muscle thickness, because we investigated the immediate effects of the MACM. Moreover, the increase in muscle thickness was insufficient to demonstrate the superiority of the MACM. Further studies are needed to determine the persistence of the effects of the MACM using various clinical tools. It is also necessary to compare the effectiveness of the MACM to other exercise methods in the patient population, which could be investigated to determine whether the MACM is a useful method to increase muscle co-contraction.

## 5. Conclusions

Our findings indicate that the MACM may be an effective and useful method compared to the ADIM in increasing the thicknesses of the TrA, IO, EO, and RA muscles. This provides clinical insight into the changes in abdominal muscle thickness during the MACM in terms of abdominal co-contraction.

## Figures and Tables

**Table 1:** General characteristics of the subjects.

Sex	Number of Subjects	Age	Height (cm)	Weight (kg)	Body Mass Index (kg/m^2^)
Male	17	23.71 (1.65)	173.18 (6.10)	72.71 (12.89)	24.17 (3.54)
Female	13	22.15 (1.46)	162.08 (5.42)	56.45 (7.87)	21.49 (2.91)

Values represent the mean (±standard deviation).

**Table 2:** Comparison of the ADIM and MACM to determine the changes in abdominal muscle thickness.

Muscles	ADIM	MACM	*p*	ES (95% CI)
TrA (cm)	0.22 (0.14)	0.36 (0.16)	<0.001 *	0.931 (0.178 to 1.685)
IO (cm)	0.24 (0.20)	0.40 (0.22)	0.001 *	0.761 (0.02 to 1.502)
EO (cm)	0.05 (0.13)	0.12 (0.20)	0.008 *	0.415 (−0.308 to 1.138)
RA (cm)	0.03 (0.12)	0.13 (0.14)	<0.001 *	0.767 (0.025 to 1.509)

ADIM: abdominal draw-in maneuver; MACM: maximum abdominal contraction maneuver; TrA: transverse abdominis; IO: internal oblique; EO: external oblique; RA: rectus abdominis; ES: effect size; values represent the mean (±standard deviation); * *p* < 0.05.

## Data Availability

Please contact the authors for data requests.
